# Antipsychotic medication adherence and preventive diabetes screening in Medicaid enrollees with serious mental illness: an analysis of real-world administrative data

**DOI:** 10.1186/s12913-020-06045-0

**Published:** 2021-01-18

**Authors:** Erica L. Stockbridge, Nathaniel J. Webb, Eleena Dhakal, Manasa Garg, Abiah D. Loethen, Thaddeus L. Miller, Karabi Nandy

**Affiliations:** 1 Department of Advanced Health Analytics and Solutions, Magellan Health, Inc, 4800 N Scottsdale Rd #4400, Scottsdale, AZ 85251 USA; 2 Department of Health Behavior & Health Systems, School of Public Health, University of North Texas Health Science Center, 3500 Camp Bowie Blvd, Fort Worth, TX 76107 USA; 3 Department of Biostatistics and Epidemiology, School of Public Health, University of North Texas Health Science Center, 3500 Camp Bowie Blvd, Fort Worth, TX 76107 USA; 4 Loopback Analytics, 14900 Landmark Blvd # 240, Dallas, TX 75254 USA; 5 Department of Population and Data Sciences, UT Southwestern Medical Center, Room E1.401A, South Campus, 5323 Harry Hines Blvd, Dallas, TX 75390 USA

**Keywords:** Serious mental illness, Mental health, Medication adherence, Diabetes screening, Healthcare quality, Medicaid, Complex comorbidity, Healthcare effectiveness data and information set (HEDIS), Social determinants of health, Claims data, Mixed effects logistic regression

## Abstract

**Background:**

There is excess amenable mortality risk and evidence of healthcare quality deficits for persons with serious mental illness (SMI). We sought to identify sociodemographic and clinical characteristics associated with variations in two 2015 Healthcare Effectiveness Data and Information Set (HEDIS) measures, antipsychotic medication adherence and preventive diabetes screening, among Medicaid enrollees with serious mental illness (SMI).

**Methods:**

We retrospectively analyzed claims data from September 2014 to December 2015 from enrollees in a Medicaid specialty health plan in Florida. All plan enrollees had SMI; analyses included continuously enrolled adults with antipsychotic medication prescriptions and schizophrenia or bipolar disorder. Associations were identified using mixed effects logistic regression models.

**Results:**

Data for 5502 enrollees were analyzed. Substance use disorders, depression, and having both schizophrenia and bipolar disorder diagnoses were associated with both HEDIS measures but the direction of the associations differed; each was significantly associated with antipsychotic medication non-adherence (a marker of suboptimal care quality) but an increased likelihood of diabetes screening (a marker of quality care). Compared to whites, blacks and Hispanics had a significantly greater risk of medication non-adherence. Increasing age was significantly associated with increasing medication adherence, but the association between age and diabetes screening varied by sex. Other characteristics significantly associated with quality variations according to one or both measures were education (associated with antipsychotic medication adherence), urbanization (relative to urban locales, residing in suburban areas was associated with both adherence and diabetes screening), obesity (associated with both adherence and diabetes screening), language (non-English speakers had a greater likelihood of diabetes screening), and anxiety, asthma, and hypertension (each positively associated with diabetes screening).

**Conclusions:**

The characteristics associated with variations in the quality of care provided to Medicaid enrollees with SMI as gauged by two HEDIS measures often differed, and at times associations were directionally opposite. The variations in the quality of healthcare received by persons with SMI that were identified in this study can guide quality improvement and delivery system reform efforts; however, given the sociodemographic and clinical characteristics’ differing associations with different measures of care quality, multidimensional approaches are warranted.

**Supplementary Information:**

The online version contains supplementary material available at 10.1186/s12913-020-06045-0.

## Background

Persons with serious mental illness (SMI), particularly schizophrenia and bipolar disorder, have a shortened lifespan relative to those without [1–6]. Up to 50% of this excess mortality is considered amenable mortality [7–10], meaning death was potentially preventable through timely and high-quality healthcare [11]. Identified disparities in the quality of care provided to persons with SMI include treatment for diabetes and hypertension, post-myocardial infarction care, and preventive care [6, 12].

Given the lower care quality and the excess mortality that may be attributable to these deficits, the care received by those with SMI is of great concern to the US Centers for Medicare and Medicaid Services (CMS) and state Medicaid agencies [13, 14]. Mental illness is common within the Medicaid population; approximately 20% of adults enrolled in Medicaid have been diagnosed with a behavioral health condition [14, 15] and Medicaid covers roughly 26% of adults in the US with SMI [16]. The majority of states require that Medicaid managed care plans use standard Healthcare Effectiveness Data and Information Set (HEDIS) quality measures developed by the National Committee for Quality Assurance to monitor enrollee care quality [15, 17].

Some HEDIS measures are specific to SMI [14]. For example, antipsychotic medications are associated with increased risk of diabetes, so many Medicaid plans assess whether adult enrollees with schizophrenia or bipolar disorder and prescriptions for antipsychotic medication have received preventive screening for diabetes during a calendar year [14, 18]. Timely diabetes care is crucial in persons with SMI; individuals with schizophrenia or bipolar disorder and diabetes are at greater risk of death due to diabetes than individuals with diabetes without a mental illness. Proactive screening enables earlier identification, treatment, and management of this chronic condition [18–20]. Another HEDIS measure focused on SMI examines whether adults with schizophrenia who take antipsychotic medication are adherent with their antipsychotic treatment regimen [21]. Non-adherence is associated with poorer mental functioning, relapse, victimization, and attendant morbidities [22–25]. Further, non-adherence complicates the treatment of SMIs, as it may be unclear whether the medication is effective or appropriately dosed [26]. Conversely, individuals with schizophrenia that regularly take antipsychotic medication have a reduced risk of schizophrenia-related hospitalizations [21, 27].

HEDIS reporting enables care quality comparisons between Medicaid managed care organizations (MCOs) and allows trends to be tracked. However, the summary information reported by MCOs does not provide insight into specific factors associated with care quality variation*.* Although antipsychotic medication adherence and diabetes screening are the two most commonly used Medicaid quality measures specifically focused on persons with schizophrenia or bipolar disorder, we identified no published multivariable analyses identifying and comparing factors associated with quality variation [14]. This knowledge gap is a concern, as Medicaid MCOs could use such information to develop programs or change processes to improve the quality of care for enrollees with these conditions. In addition, CMS has called for states to design innovative service delivery systems for persons with SMI, and information about the factors associated with care quality variations might help guide these efforts [13, 28]. To fill this gap, we used real-world administrative data to identify the sociodemographic and clinical characteristics associated with two quality of care measures: non-receipt of diabetes screening in Medicaid enrollees with schizophrenia or bipolar disorder who are taking antipsychotic medication, and antipsychotic medication non-adherence in Medicaid enrollees with schizophrenia.

## Methods

The Office of Research Compliance at the University of North Texas Health Science Center determined on behalf of the North Texas Regional Institutional Review Board that these analyses do not meet the definition of human subjects research.

### Data source

We analyzed enrollment data and medical and pharmacy claims from Magellan Complete Care (MCC) of Florida for services rendered September 2014 through December 2015. MCC of Florida was a Medicaid specialty health plan designed specifically for persons with SMI, with 42,138 enrollees as of December 2015 [29]. The Florida Agency for Health Care Administration determined eligibility for the plan based on medical or pharmacy claims data from services occurring prior to enrollment in the plan. To be eligible, an individual must have had a prior diagnosis of bipolar disorder, schizophrenia, major depression, obsessive-compulsive disorder, or another psychotic or delusional disorder, or the person must have received a prescription for a medication used to treat these disorders [30]. Analyzed data represents 40 of Florida’s 67 counties, including Miami-Dade and Broward.

We included enrollee data in analyses if HEDIS-defined criteria for antipsychotic medication non-adherence and/or non-receipt of diabetes preventive screening were met. While inclusion criteria for the two measures differed slightly, all had a diagnosis of schizophrenia or bipolar disorder, one or more oral or injectable medical or pharmacy claims for antipsychotic medications (Additional file 1), and remained continuously enrolled in the health plan with no more than one gap of less than 45 days.

### Measures

#### Outcome variables

Outcome variables were created using standard, validated HEDIS logic for the 2015 measurement year; they examine healthcare utilization occurring during 2015. Specific details, including International Classification of Disease (ICD) codes, Healthcare Common Procedure Coding System (HCPCS) codes, Current Procedural Terminology (CPT) codes [31], and national drug codes (NDC), are available elsewhere [32].

#### Antipsychotic medication non-adherence

Antipsychotic medication non-adherence was assessed for enrollees ages 19 to 64 years, inclusive, using the logic for the HEDIS measure “Adherence to Antipsychotic Medications for Individuals with Schizophrenia (SAA)” [21]. Only members with two or more antipsychotic medication dispensing events in 2015 were included. Adherence was defined as remaining on antipsychotic medication for at least 80% of the period between the first and last dispensing event. Specifically, this variable examines the proportion of days covered by an antipsychotic medication prescription during this period; persons with ≥80% of days covered were deemed adherent.

#### Non-receipt of preventive diabetes screening

Logic for the HEDIS measure “Diabetes Screening for People with Schizophrenia or Bipolar Disorder Who Are Using Antipsychotic Medications (SSD)” was used to create the non-receipt of preventive diabetes screening variable for enrollees aged 19 to 64 years, inclusive, without diabetes [21]. We determined whether preventive diabetes screening with a glucose or hemoglobin A1c test was received during 2015 by enrollees dispensed an antipsychotic medication at least one time in 2015 and who had schizophrenia spectrum or bipolar disorders.

#### Explanatory variables

Andersen’s Behavioral Model of Health Services Use guided our selection of explanatory variables; this model suggests that individual healthcare utilization is determined by predisposing characteristics, enabling resources, and clinical need [33]. Data to create explanatory variables were extracted from enrollment data or generated from members’ physical locations or claims for services rendered September 2014 through December 2015. Predisposing characteristics included sex, age, race/ethnicity, and education levels [33, 34]. Enabling resources included language (dichotomized), urbanization of members’ counties [35], whether a county was a designated geographic mental health professional shortage area (MH HPSA) [36], and the distance between each member’s residence and his/her primary care physician (PCP) based on zip code [33, 34]. We used Optum Impact Pro’s diagnosis-related clinical indicators from claims for services rendered between September 2014 and December 2015 to identify members’ clinical needs [37, 38]. Aside from SMI, clinical/comorbid variables included depression, anxiety, substance use disorder (including alcohol and non-alcohol substances), asthma, cardiac conditions (including congestive heart failure, coronary artery disease, and myocardial infarction), chronic obstructive pulmonary disease, hypertension, obesity, and diabetes.

An additional variable was created to reflect whether a member had been diagnosed with schizophrenia only, bipolar only, or both bipolar and schizophrenia. This was used when examining the receipt of diabetes screening per HEDIS logic. Similarly, a bipolar disorder covariate was created for use in adherence to antipsychotic medication analyses. As HEDIS definitions required that all members included in that analysis be diagnosed with schizophrenia, this outcome variable reflects whether a member had both schizophrenia and bipolar diagnoses or received a schizophrenia diagnosis but no bipolar diagnosis.

### Statistical methods

We determined how many enrollees met criteria for inclusion into one or both of the HEDIS outcome variables and used mixed effects logistic regression models for all statistical analyses. Because some health plan enrollees had the same PCP, we controlled for PCP as a random effect in these models. We used a series of bivariate mixed effects logistic regression models to examine the unadjusted relationship between explanatory and outcome variables. Age by sex interactions were tested and interactions that were non-significant in post-hoc analyses were excluded.

We used two multivariable mixed effects logistic regression models to estimate adjusted associations between outcome and explanatory variables. The first included data only for those eligible for the antipsychotic medication adherence analysis; the second included data only for those eligible for the diabetes screening measure analysis. Average predicted probabilities of each outcome were calculated for each category of the explanatory variables to provide insight into the practical significance of findings from both multivariable models. The age by sex interactions were illustrated with graphs of these probabilities. All statistical tests were two-sided, significance was tested at *p* < 0.05, and analyses were conducted using Stata 14.2 [StataCorp, College Station, TX].

## Results

A total of 5502 Medicaid specialty plan enrollees were included in our analyses. Of these, 3705 (67.3%) met the criteria for inclusion in the HEDIS measure examining adherence to antipsychotic medication, 4910 (89.2%) met the criteria for inclusion in the HEDIS measure examining diabetes testing, and 3113 (56.6%) were eligible for inclusion in both measures.

### Measure 1: non-adherence to antipsychotic medication

Of 3705 enrollees with schizophrenia and two or more antipsychotic medication dispensing events in 2015, 1778 (48%) were not adherent to medication (Table 1).

**Table 1:** Characteristics of Medicaid specialty plan enrollees with schizophrenia or bipolar disorders who are on antipsychotic medications, examined in the context of two HEDIS measures, 2015. The two measures of interest were 1) adherence to antipsychotic medication (SAA), which includes persons with schizophrenia, and 2) receipt of recommended diabetes screening (SSD), which includes persons with schizophrenia or bipolar disorder. Significance was tested using unadjusted random effect logistic regression models

Medicaid Enrollee Characteristics	Measure 1: Adherence to Antipsychotic Medication (SAA)				Measure 2: Receipt of Diabetes Screening (SSD)			
	Total in SAA Denominator N=3705 n (%)	Adherent *N*=1927 n (%)	Non-Adherent *N*=1778 n (%)	*p*-value	Total in SSD Denominator N=4910 n (%)	Screened *N*=3487 n (%)	Not Screened *N*=1423 n (%)	*p*-value
Sex								
Female	1461 (39.43)	752 (39.02)	709 (39.88)	0.699	2327 (47.39)	1780 (51.05)	547 (38.44)	< 0.001
Male	2244 (60.57)	1175 (60.98)	1069 (60.12)		2583 (52.61)	1707 (48.95)	876 (61.56)	
Age Group								
18–29	n/a	n/a	n/a	< 0.001	1153 (23.48)	771 (22.11)	382 (26.84)	< 0.001
19–29	726 (19.60)	296 (15.36)	430 (24.18)		n/a	n/a	n/a	
30–39	875 (23.62)	423 (21.95)	452 (25.42)		1263 (25.72)	864 (24.78)	399 (28.04)	
40–49	755 (20.38)	391 (20.29)	364 (20.47)		1003 (20.43)	725 (20.79)	278 (19.54)	
50–64	1349 (36.41)	817 (42.40)	532 (29.92)		1491 (30.37)	1127 (32.32)	364 (25.58)	
Race/Ethnicity								
White	782 (21.11)	485 (25.17)	297 (16.70)	< 0.001	1451 (29.55)	1075 (30.83)	376 (26.42)	< 0.001
Black / African American	1324 (35.74)	594 (30.83)	730 (41.06)		1415 (28.82)	943 (27.04)	472 (33.17)	
Hispanic	575 (15.52)	333 (17.28)	242 (13.61)		723 (14.73)	540 (15.49)	183 (12.86)	
Other	83 (2.24)	59 (3.06)	24 (1.35)		98 (2.00)	70 (2.01)	28 (1.97)	
Not Provided	941 (25.40)	456 (23.66)	485 (27.28)		1223 (24.91)	859 (24.63)	364 (25.58)	
Language								
English	3054 (82.43)	1529 (79.35)	1525 (85.77)	< 0.001	4211 (85.76)	2977 (85.37)	1234 (86.72)	0.351
Not English	651 (17.57)	398 (20.65)	253 (14.23)		699 (14.24)	510 (14.63)	189 (13.28)	
Urbanicity								
Large Central Metro (most urban)	2043 (55.14)	1044 (54.18)	999 (56.19)	0.217	2575 (52.44)	1804 (51.74)	771 (54.18)	0.094
Large Fringe Metro	936 (25.26)	504 (26.15)	432 (24.30)		1231 (25.07)	922 (26.44)	309 (21.71)	
Medium Metro	602 (16.25)	307 (15.93)	295 (16.59)		891 (18.15)	619 (17.75)	272 (19.11)	
Small Metro or Non-Metro (most rural)	124 (3.35)	72 (3.74)	52 (2.92)		213 (4.34)	142 (4.07)	71 (4.99)	
Education Levels in County								
15%+ of adults have HS degree	2407 (64.97)	1222 (63.41)	1185 (66.65)	0.131	3307 (67.35)	2319 (66.50)	988 (69.43)	0.030
< 15% of adults have HS degree	1298 (35.03)	705 (36.59)	593 (33.35)		1603 (32.65)	1168 (33.50)	435 (30.57)	
County is Mental Health HPSA								
No	3488 (94.14)	1819 (94.40)	1669 (93.87)	0.714	4577 (93.22)	3257 (93.40)	1320 (92.76)	0.880
Yes	217 (5.86)	108 (5.60)	109 (6.13)		333 (6.78)	230 (6.60)	103 (7.24)	
Member’s Distance from PCP								
Same Zip Code	788 (21.27)	406 (21.07)	382 (21.48)	0.154	1012 (20.61)	703 (20.16)	309 (21.71)	0.248
> 0 to < 5 Miles	1274 (34.39)	695 (36.07)	579 (32.56)		1621 (33.01)	1148 (32.92)	473 (33.24)	
5 to < 15 Miles	1170 (31.58)	586 (30.41)	584 (32.85)		1592 (32.42)	1129 (32.38)	463 (32.54)	
15 to < 30 Miles	316 (8.53)	166 (8.61)	150 (8.44)		436 (8.88)	314 (9.00)	122 (8.57)	
>=30 Miles	157 (4.24)	74 (3.84)	83 (4.67)		249 (5.07)	193 (5.53)	56 (3.94)	
Depression								
No	2430 (65.59)	1343 (69.69)	1087 (61.14)	< 0.001	3144 (64.03)	1958 (56.15)	1186 (83.35)	< 0.001
Yes	1275 (34.41)	584 (30.31)	691 (38.86)		1766 (35.97)	1529 (43.85)	237 (16.65)	
Anxiety								
No	2358 (63.64)	1293 (67.10)	1065 (59.90)	< 0.001	2919 (59.45)	1794 (51.45)	1125 (79.06)	< 0.001
Yes	1347 (36.36)	634 (32.90)	713 (40.10)		1991 (40.55)	1693 (48.55)	298 (20.94)	
Bipolar Disorder (in addition to Schizophrenia)								
No	2856 (77.09)	1571 (81.53)	1285 (72.27)	< 0.001	n/a	n/a	n/a	n/a
Yes	849 (22.91)	356 (18.47)	493 (27.73)		n/a	n/a	n/a	
Schizophrenia or Bipolar Disorder								
Bipolar	n/a	n/a	n/a	n/a	1409 (28.70)	1021 (29.28)	388 (27.27)	< 0.001
Schizophrenia	n/a	n/a	n/a		2654 (54.05)	1717 (49.24)	937 (65.85)	
Both Schizophrenia and Bipolar	n/a	n/a	n/a		847 (17.25)	749 (21.48)	98 (6.89)	
Substance Use Disorder								
No	2210 (59.65)	1285 (66.68)	925 (52.02)	< 0.001	2795 (56.92)	1695 (48.61)	1100 (77.30)	< 0.001
Yes	1495 (40.35)	642 (33.32)	853 (47.98)		2115 (43.08)	1792 (51.39)	323 (22.70)	
Asthma								
No	2802 (75.63)	1488 (77.22)	1314 (73.90)	0.019	3639 (74.11)	2400 (68.83)	1239 (87.07)	< 0.001
Yes	903 (24.37)	439 (22.78)	464 (26.10)		1271 (25.89)	1087 (31.17)	184 (12.93)	
Cardiac Condition (CHF/CAD/MI)								
No	3622 (97.76)	1892 (98.18)	1730 (97.30)	0.157	4841 (98.59)	3422 (98.14)	1419 (99.72)	< 0.001
Yes	83 (2.24)	35 (1.82)	48 (2.70)		69 (1.41)	65 (1.86)	4 (0.28)	
COPD								
No	3166 (85.45)	1637 (84.95)	1529 (86.00)	0.512	4269 (86.95)	2921 (83.77)	1348 (94.73)	< 0.001
Yes	539 (14.55)	290 (15.05)	249 (14.00)		641 (13.05)	566 (16.23)	75 (5.27)	
Hypertension								
No	1867 (50.39)	977 (50.70)	890 (50.06)	0.667	2829 (57.62)	1707 (48.95)	1122 (78.85)	< 0.001
Yes	1838 (49.61)	950 (49.30)	888 (49.94)		2081 (42.38)	1780 (51.05)	301 (21.15)	
Obesity								
No	3062 (82.65)	1572 (81.58)	1490 (83.80)	0.074	4260 (86.76)	2893 (82.97)	1367 (96.06)	< 0.001
Yes	643 (17.35)	355 (18.42)	288 (16.20)		650 (13.24)	594 (17.03)	56 (3.94)	
Diabetes								
No	2922 (78.87)	1474 (76.49)	1448 (81.44)	< 0.001	n/a	n/a	n/a	n/a
Yes	783 (21.13)	453 (23.51)	330 (18.56)		n/a	n/a	n/a	

**Fig. 1:**
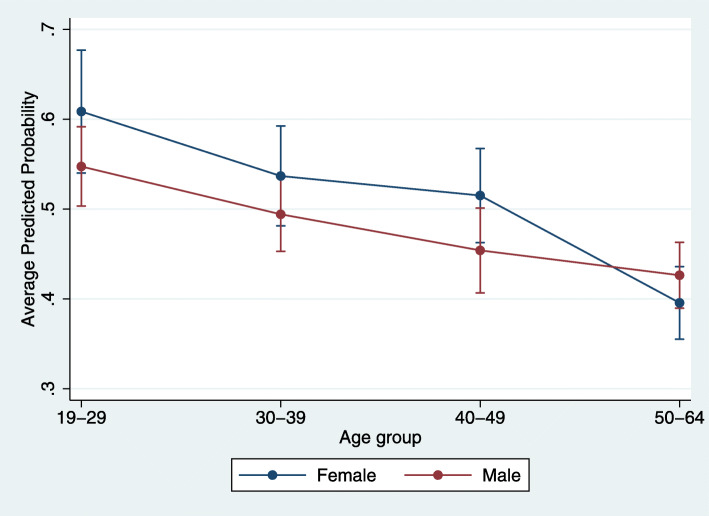
Average predicted probabilities of non-adherence to antipsychotic medication by age and sex. Sample includes Medicaid enrollees ages 19 to 64 years with schizophrenia spectrum disorders (*n*=3705). The interaction between age and sex was nonsignificant, but there was a significant association between age and non-adherence. Based on full binomial logistic regression model 1; detailed model results are available in Table 2

Tables 1 and 2 and Fig. 1 detail descriptive characteristics and results relative to medication non-adherence among enrollees diagnosed with schizophrenia. Notable significant predisposing associations to non-adherence include age 18–29, Hispanic race/ethnicity, large central metro residence, residence in counties with higher education levels, and being non-English speaking; notable comorbid health conditions significantly associated with non-adherence included depression, dual bipolar and schizophrenia diagnoses, and substance use disorder (Tables 1 and 2). Enrollees with either diabetes or obesity had significantly lower odds of non-adherence compared to those without (Tables 1 and 2). No significant age by sex interaction was identified (Table 2 and Fig. 1).

**Table 2:** Mixed effect logistic regression model results examining two HEDIS measures in Medicaid enrollees with schizophrenia or bipolar disorders who are on antipsychotic medications, 2015. Model 1 examines characteristics associated with non-adherence to antipsychotic medication in persons with schizophrenia (SAA; *n*=3705). Model 2 examines characteristics associated with not receiving recommended diabetes screening in persons with schizophrenia or bipolar disorder (SSD; *n*=4910). Higher odds represent a higher likelihood of poor care quality as evaluated by the measure

Medicaid Enrollee Characteristics	Model 1: Non-Adherence to Antipsychotic Medication (SAA)			Model 2: Not Receiving Diabetes Screening (SSD)		
	Odds Ratio (95% CI)	*p*-value	Predicted Probability (95% CI)	Odds Ratio (95% CI)	*p*-value	Predicted Probability (95% CI)
Sex						
Female	1.00 (base)	n/a	n/a	1.00 (base)	n/a	n/a
Male	0.77 (0.54, 1.08)	0.133	n/a	1.74 (1.28, 2.37)	< 0.001	n/a
Age Group						
18–29	n/a	n/a	n/a	1.00 (base)	n/a	n/a
19–29	1.00 (base)	n/a	n/a	n/a	n/a	n/a
30–39	0.73 (0.50, 1.07)	0.106	n/a	1.18 (0.85, 1.63)	0.322	n/a
40–49	0.67 (0.46, 0.97)	0.035	n/a	1.31 (0.93, 1.82)	0.118	n/a
50–64	0.40 (0.28, 0.57)	< 0.001	n/a	1.27 (0.91, 1.77)	0.156	n/a
Age*Sex Interaction						
18–29 Female	n/a	n/a	n/a	1.00 (base)	n/a	0.233 (0.195, 0.271)
18–29 Male	n/a	n/a	n/a	1.00 (base)	n/a	0.321 (0.289, 0.353)
19–29 Female	1.00 (base)	n/a	0.609 (0.540, 0.677)	n/a	n/a	n/a
19–29 Male	1.00 (base)	n/a	0.547 (0.503, 0.592)	n/a	n/a	n/a
30–39 Female	1.00 (base)	n/a	0.537 (0.481, 0.592)	1.00 (base)	n/a	0.258 (0.223, 0.292)
30–39 Male	1.09 (0.69, 1.71)	0.710	0.494 (0.453, 0.536)	0.84 (0.56, 1.26)	0.393	0.319 (0.286, 0.351)
40–49 Female	1.00 (base)	n/a	0.515 (0.463, 0.568)	1.00 (base)	n/a	0.274 (0.237, 0.310)
40–49 Male	1.01 (0.64, 1.59)	0.978	0.454 (0.407, 0.501)	0.67 (0.43, 1.03)	0.068	0.298 (0.259, 0.337)
50–64 Female	1.00 (base)	n/a	0.396 (0.355, 0.436)	1.00 (base)	n/a	0.269 (0.236, 0.302)
50–64 Male	1.49 (0.99, 2.26)	0.058	0.426 (0.390, 0.463)	0.55 (0.37, 0.82)	0.003	0.262 (0.231, 0.293)
Race/Ethnicity						
White	1.00 (base)	n/a	0.379 (0.344, 0.413)	1.00 (base)	n/a	0.284 (0.260, 0.309)
Black / African American	2.14 (1.76, 2.60)	< 0.001	0.555 (0.527, 0.582)	1.04 (0.85, 1.27)	0.716	0.290 (0.267, 0.314)
Hispanic	1.30 (1.01, 1.68)	0.039	0.439 (0.395, 0.484)	0.89 (0.68, 1.16)	0.392	0.266 (0.232, 0.300)
Other	0.84 (0.50, 1.40)	0.494	0.340 (0.237, 0.444)	0.80 (0.48, 1.34)	0.400	0.250 (0.176, 0.324)
Not Provided	1.64 (1.33, 2.02)	< 0.001	0.493 (0.461, 0.525)	0.99 (0.81, 1.23)	0.962	0.283 (0.259, 0.308)
Language						
English	1.00 (base)	n/a	0.481 (0.463, 0.499)	1.00 (base)	n/a	0.289 (0.274, 0.304)
Not English	0.97 (0.79, 1.21)	0.803	0.474 (0.430, 0.518)	0.76 (0.60, 0.97)	0.025	0.246 (0.213, 0.280)
Urbanicity						
Large Central Metro (most urban)	1.00 (base)	n/a	0.501 (0.478, 0.524)	1.00 (base)	n/a	0.292 (0.272, 0.313)
Large Fringe Metro	0.79 (0.66, 0.94)	0.010	0.447 (0.414, 0.480)	0.74 (0.60, 0.91)	0.004	0.245 (0.219, 0.270)
Medium Metro	0.89 (0.72, 1.11)	0.307	0.476 (0.434, 0.517)	1.02 (0.80, 1.30)	0.855	0.296 (0.264, 0.328)
Small Metro or Non-Metro (most rural)	0.61 (0.39, 0.97)	0.036	0.390 (0.294, 0.486)	1.24 (0.79, 1.94)	0.351	0.327 (0.256, 0.398)
Education Levels in County						
15%+ of adults have HS degree	1.00 (base)	n/a	0.495 (0.474, 0.516)	1.00 (base)	n/a	0.290 (0.273, 0.307)
< 15% of adults have HS degree	0.83 (0.70, 0.97)	0.024	0.451 (0.422, 0.480)	0.86 (0.71, 1.05)	0.134	0.267 (0.242, 0.292)
County is Mental Health HPSA						
No	1.00 (base)	n/a	0.475 (0.458, 0.492)	1.00 (base)	n/a	0.283 (0.268, 0.298)
Yes	1.41 (0.99, 2.03)	0.060	0.554 (0.475, 0.633)	0.96 (0.66, 1.39)	0.810	0.276 (0.219, 0.332)
Patient’s Distance from PCP						
Same Zip Code	1.00 (base)	n/a	0.494 (0.460, 0.528)	1.00 (base)	n/a	0.279 (0.253, 0.304)
> 0 to < 5 Miles	0.86 (0.72, 1.04)	0.128	0.460 (0.433,0.488)	1.03 (0.85, 1.26)	0.746	0.284 (0.262, 0.306)
5 to < 15 Miles	0.98 (0.81, 1.19)	0.872	0.490 (0.462, 0.519)	1.06 (0.87, 1.30)	0.550	0.289 (0.266, 0.311)
15 to < 30 Miles	0.86 (0.65, 1.14)	0.283	0.459 (0.404, 0.514)	1.03 (0.77, 1.39)	0.822	0.284 (0.243, 0.325)
>=30 Miles	1.13 (0.78, 1.64)	0.507	0.523 (0.445, 0.601)	0.80 (0.55, 1.16)	0.239	0.244 (0.192, 0.296)
Depression						
No	1.00 (base)	n/a	0.465 (0.444, 0.486)	1.00 (base)	n/a	0.309 (0.292, 0.326)
Yes	1.21 (1.02, 1.43)	0.028	0.508 (0.478, 0.539)	0.53 (0.44, 0.64)	< 0.001	0.210 (0.186, 0.234)
Anxiety						
No	1.00 (base)	n/a	0.480 (0.458, 0.501)	1.00 (base)	n/a	0.303 (0.286, 0.321)
Yes	1.00 (0.84, 1.18)	0.973	0.479 (0.449, 0.509)	0.65 (0.54, 0.78)	< 0.001	0.235 (0.211, 0.258)
Bipolar Disorder (in addition to Schizophrenia)						
No	1.00 (base)	n/a	0.463 (0.444, 0.482)	n/a	n/a	n/a
Yes	1.38 (1.15, 1.65)	0.001	0.537 (0.500, 0.573)	n/a	n/a	n/a
Schizophrenia or Bipolar Disorder						
Bipolar	n/a	n/a	n/a	1.00 (base)	n/a	0.308 (0.283, 0.334)
Schizophrenia	n/a	n/a	n/a	0.89 (0.75, 1.07)	0.224	0.290 (0.272, 0.308)
Both Schizophrenia and Bipolar	n/a	n/a	n/a	0.45 (0.34, 0.59)	< 0.001	0.189 (0.157, 0.220)
Substance Use Disorder						
No	1.00 (base)	n/a	0.436 (0.414, 0.458)	1.00 (base)	n/a	0.330 (0.311, 0.348)
Yes	1.59 (1.36, 1.86)	< 0.001	0.544 (0.517, 0.571)	0.44 (0.37, 0.52)	< 0.001	0.198 (0.178, 0.218)
Asthma						
No	1.00 (base)	n/a	0.471 (0.452, 0.490)	1.00 (base)	n/a	0.296 (0.280, 0.312)
Yes	1.16 (0.96, 1.40)	0.113	0.506 (0.470, 0.542)	0.64 (0.52, 0.78)	< 0.001	0.226 (0.200, 0.254)
Cardiac Condition						
No	1.00 (base)	n/a	0.476 (0.459, 0.493)	1.00 (base)	n/a	0.284 (0.270, 0.298)
Yes	1.30 (0.96, 1.76)	0.096	0.536 (0.468, 0.603)	0.62 (0.35, 1.10)	0.103	0.213 (0.134, 0.291)
COPD						
No	1.00 (base)	n/a	0.484 (0.467, 0.502)	1.00 (base)	n/a	0.284 (0.270, 0.299)
Yes	0.87 (0.69, 1.10)	0.224	0.451 (0.404, 0.499)	0.85 (0.63, 1.16)	0.300	0.259 (0.213, 0.305)
Diabetes						
No	1.00 (base)	n/a	n/a	n/a	n/a	n/a
Yes	0.74 (0.62, 0.89)	0.002	n/a	n/a	n/a	n/a
Hypertension						
No	1.00 (base)	n/a	0.472 (0.448, 0.497)	1.00 (base)	n/a	0.329 (0.311, 0.348)
Yes	1.07 (0.91, 1.25)	0.438	0.487 (0.462, 0.512)	0.43 (0.36, 0.51)	< 0.001	0.194 (0.174, 0.214)
Obesity						
No	1.00	n/a	0.489 (0.471, 0.507)	1.00 (base)	n/a	0.301 (0.286, 0.317)
Yes	0.79 (0.65, 0.95)	0.014	0.434 (0.395, 0.473)	0.26 (1.19, 0.35)	< 0.001	0.121 (0.928, 0.150)

### Measure 2: non-receipt of preventive diabetes screening

Of the 4910 Medicaid specialty plan enrollees with schizophrenia or bipolar disorder who were dispensed an antipsychotic medication at least one time in 2015, 1423 (29%) did not receive preventive diabetes screening (Table 1).

**Fig. 2:**
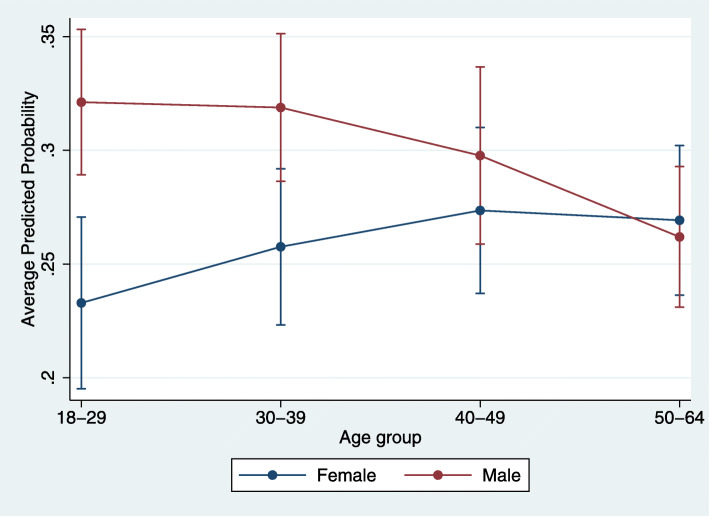
Average predicted probabilities of not receiving preventive diabetes screening by age and sex. Sample includes Medicaid enrollees ages 18 to 64 years with schizophrenia spectrum or bipolar disorder (*n*=4910). There was a significant interaction between age and sex. Based on full binomial logistic regression model 2; detailed model results are available in Table 2

Tables 1 and 2 detail descriptive characteristics and results relative to non-receipt of diabetes screening among enrollees receiving antipsychotic medications. Notable significant characteristics associated with non-receipt include male sex and large central metro residence. Enrollees with depression, anxiety, both bipolar and schizophrenia diagnoses (compared to having bipolar only), substance use disorder, asthma, hypertension, and obesity had significantly lower odds of non-receipt compared to those without (Tables 1 and 2). A significant age by sex interaction indicated that males across all age groups had a higher average predicted likelihood of not receiving diabetes screening compared to their female counterparts except in the 50–64 age group, wherein females had higher average predicted probability of not receiving diabetes screening compared to their male counterparts (Table 2 and Fig. 2).

## Discussion

We identified sociodemographic and clinical characteristics associated with antipsychotic medication adherence and diabetes screening among Florida Medicaid enrollees with SMI. Despite substantial overlap between the measures in terms of persons included (i.e., 56.6% of those eligible for inclusion in either measure were eligible for both measures) the sociodemographic and clinical characteristics associated with variations in care quality often differed.

### Clinical characteristics

Our findings related to substance use disorders, depression, and having both schizophrenia and bipolar disorder diagnoses were most notable, as these variables were significantly associated with both HEDIS measures, but in different directions. Each was associated with both antipsychotic medication non-adherence (a marker of suboptimal care quality) and an increased likelihood of receiving recommended diabetes screening (a marker of higher quality care) (Table 2). Health plans may conduct proactive, targeted outreach to patients who are at risk of not receiving quality care as measured by HEDIS care quality standards, so the contrasting direction of these associations may present a challenge from a population health management perspective. Our results suggest that measures of quality cannot be considered collectively, as predictive algorithms may identify the same patient as being at increased risk of poor care quality as assessed by one measure, but decreased risk based on another.

Despite their seeming contradictions these findings are well-aligned with past research, including reports that Medicaid enrollees with SMI and a comorbid substance use disorder diagnosis are both more likely to receive diabetes screening [39] and less adherent to antipsychotic medications [40–50]. Similarly, our findings concur with reported associations between depressive symptoms and non-adherence to antipsychotic medications in persons with schizophrenia [51–53]. Conversely, associations between depression diagnoses or symptomatology and diabetes testing in persons with schizophrenia or bipolar disorder who use antipsychotic medications appear understudied. Our methods cannot elicit provider motivations, but it is plausible that the observed associations relate to heightened concerns about metabolic syndrome [54]. Alternatively, persons with depression diagnoses may have more healthcare encounters [55–57], thus increasing opportunity for screening [39].

Associations between concurrently diagnosed bipolar disorder and schizophrenia and either antipsychotic medication adherence or diabetes testing also appear understudied. This gap could arise from the view that mood disorders and schizophrenia spectrum disorders are non-concurrent conditions [58]. Although patients may simultaneously experience symptoms of both schizophrenia and mood disorder (e.g., mania, depressed mood), diagnostic coding and clinical practices dictate that these persons receive a schizophrenia spectrum disorder diagnosis (specifically, schizoaffective disorder) rather than separate mood and schizophrenia diagnoses [58, 59]. In practice both types of diagnoses may appear concurrently in real-world administrative data due to multiple diagnosing providers or variations and imperfections in coding practices [58, 60]. Although many studies stringently categorize patients into mutually exclusive diagnostic groups (i.e., all patients with a schizophrenia spectrum diagnosis are categorized as having schizophrenia and only those without a schizophrenia diagnosis are categorized as having a mood disorder) [19, 61–63], such methods mask clinical complexity for patients whose real-world data include both types of conditions. This lower fidelity is a loss to health plans, accountable care organizations, and other organizations with population health initiatives that rely on administrative data. In addition to our findings, other researchers have identified that patients whose administrative data include both bipolar and schizophrenia diagnoses have higher prevalence of comorbid substance use disorders and chronic medical conditions, higher likelihood of using antipsychotic medications, higher healthcare utilization rates, and higher healthcare costs [58].

Hypertension, asthma, and anxiety were each significantly associated with an increased likelihood of diabetes screening (Table 2). The association between hypertension and diabetes screening has been well-established [19, 39, 64–67], but we found no studies examining comorbid asthma or anxiety and diabetes screening in populations similar to ours; our identification of these positive associations adds to the literature (Table 2). We also identified no previous work exploring associations between antipsychotic medication adherence and hypertension or asthma. In contrast, studies examining antipsychotic medication adherence and anxiety have yielded conflicting findings; one found that anxiety was associated with non-adherence [41], while another mirrored our study in identifying no significant association between anxiety and adherence [51].

Diabetes and obesity were each independently associated with an increased likelihood of antipsychotic medication adherence (Table 2). Our findings related to adherence and obesity differ directionally from previous research; when persons with schizophrenia were surveyed, obese respondents were more likely to report being non-adherent compared to those with normal-range BMIs [68]. In another study, 58.5% of persons with bipolar disorder who were non-adherent to antipsychotic medication reported that weight gain was one reason for non-adherence [69]. Even so, our findings are consistent with the fact that antipsychotic medication use is associated with a risk of obesity and diabetes [70–72]. Further, the risk of diabetes for some antipsychotic medications appears to be dose-dependent – as the dose increases, the likelihood of diabetes increases [72]. Given those findings, the associations that we observed are as expected; adherence may be contributing to obesity and/or diabetes. Further research is needed to examine causality and better understand why past studies on self-reported adherence and weight yielded findings different from ours.

### Predisposing socio-demographic characteristics

The associations between care quality and age and sex also differed for the two measures. Antipsychotic medication adherence increased with age independent of sex (Table 2 and Fig. 1), but sex modified the association between age and diabetes screening (Table 2 and Fig. 2). Increasing age is associated with increasing adherence in many prior studies, perhaps due to increasing awareness of the need for medication [40, 46, 69, 73–77]. Older patients have more experience with negative consequences of non-adherence, including relapses and hospitalizations [78]. Conversely, there have been inconsistent findings regarding associations between sex and diabetes screening; often no association is identified, though age by sex interactions are typically not tested [19, 64, 65, 79]. We found that at younger ages women were more likely to receive screening than men, but these differences disappeared as age increased (Table 2 and Fig. 2). It is plausible that the typically higher rate of healthcare service use among young women provides greater opportunity to receive diabetes screening until sex differences in utilization rates (and thus screening opportunities) narrow with age [80].

### Enabling socio-demographic characteristics

We observed a relatively higher likelihood of diabetes screening in non-English speakers (Table 2). This is surprising given that persons in the US who speak a language other than English may face structural barriers when seeking healthcare, and on a national level there are marked disparities in healthcare access for non-English speakers [81, 82]. Florida’s demographics likely contribute to this finding -- there are many Spanish-speaking immigrants in Florida and, compared to other parts of the US, also many Spanish-speaking healthcare providers [83, 84]. We could not examine patient-provider language concordance or evaluate causality, but it is possible that the relative availability of providers who speak a language other than English in Florida may have increased the likelihood of diabetes testing in like enrollees, as patient-provider language concordance facilitates care quality [85, 86].

Our findings that blacks and Hispanics had a higher likelihood of antipsychotic non-adherence relative to whites underscore concerns that minority patients face disparate healthcare quality (Table 2) [40, 41, 75, 87, 88]. This suggests that there are opportunities for Medicaid MCOs to improve the quality of healthcare for minority enrollees with SMI; evidence-based interventions that improve medication adherence and care quality in minority populations (e.g., reminder systems, provider education, direct-to-patient services) may be warranted [89]. Interventions which target patient attitudes and beliefs within minority enrollees may also be beneficial, as blacks are more likely than whites to report a fear of addiction and express the belief that medication is a symbol of illness [90]. Further, change at a societal level may be required to lessen the implicit racial biases of providers that likely contribute to healthcare quality disparities such as those observed in the current study [91].

### Implications for practice

Our findings indicate that the characteristics associated with variations in the quality of care provided to Medicaid enrollees with SMI as gauged by two HEDIS measures often differed. This suggests that multidimensional approaches to improving the care quality of persons with SMI are warranted, as different mechanisms may be at play to different degrees depending on how quality is measured. While one might assume that provider factors primarily drive preventive screening (because providers conduct testing) whereas health plan enrollee factors drive medication adherence (because enrollees take the medications), the reality is more complex. Both enrollee and provider factors drive both of these measures, so strategies to influence these measures must target both groups to maximize impact.

Provider and system changes are generally the focus of initiatives to increase the rates of preventive screening. System changes, including the co-location of mental and physical health care services, show promise in increasing diabetes screening in persons with SMI [92, 93]. Additionally, providers are the focus of recent quality-focused pay for performance and reimbursement reform initiatives which show promise in improving preventive screening [94]. However, patients also play a role in obtaining preventive services, so payers might consider additional enrollee-focused strategies. For example, initiatives to change patients’ attitudes regarding health responsibilities and benefits may improve preventive diabetes screening rates [95]. When it comes to improving medication adherence, patient-focused strategies such as counseling and mobile text messaging are common [96]. However, physicians play a critical role in medication adherence. The American Medical Association recognizes the role that physicians play in facilitating medication adherence and provides continuing education focused on adherence [97]. Adherence is greater for patients of providers who provide person-centered care that results in agreement about the presenting problem and how to manage that problem [98].

Clearly health plans’ approaches to improving care quality will be most effective when strategies focus on the behavior and attitudes of both providers and enrollees. Our findings will enable plans to focus such initiatives on enrollees with SMI at especially high risk of poor care quality and on the providers serving these enrollees. This will be of special interest to Medicaid plans and state Medicaid agencies as the largest payer of mental health services in the US [99].

### Limitations & opportunities for future research

While our study provides important new insights about variations in the quality of care provided to Medicaid enrollees with SMI, there are limitations. We only analyzed information contained within administrative data, so some drivers of care quality and behavior (e.g., patient attitudes, perceived benefits, and insight into illness [100]) could not be studied. While our total sample size was robust, only a small number of persons were in race/ethnicity groups other than white, black, or Hispanic. Further, roughly a quarter of persons in our study had an unspecified race/ethnicity; we retained these individuals in analyses by including an “unknown” race/ethnicity category. It is unknown if our results regarding this variable would change if these data had been available. Our study focus was narrow, with data limited to sociodemographic and clinical characteristics of enrollees in a single SMI specialty plan in Florida. Future studies should seek to confirm the generalizability of our findings while including measures of enrollees’ healthcare utilization and concurrent medication use. Still, we provide important insights about the quality of care for enrollees of this relatively new type of Medicaid managed care plan, as similar “vertical carve-out” Medicaid plans are becoming increasingly common [101, 102].

Opportunities to build on the current study include expanding on the HEDIS-defined measures of preventive diabetes screening and antipsychotic medication adherence. The antipsychotic medication adherence measure only includes persons with schizophrenia, but the adherence of persons taking antipsychotics for other conditions may be of interest. Additionally, these measures do not include persons older than 64, and the lower bounds of the eligible age ranges differ for the two measures. Despite these inconsistencies, HEDIS definitions are validated, standard, and widely used. As such, our methods can be replicated, and the overall quality rates can be compared to those of other Medicaid managed care plans [18, 21].

Our observational, cross-sectional data disallow us from making causal inferences, and potential explanations for our findings should be considered speculative. Further, the analyses were exploratory; our findings are data-driven. Even so, our results create a foundation for future hypothesis-driven research. Although claims data provide important information about the medical and behavioral health of enrollees, these data only include diagnoses associated with healthcare services. The data do not include undiagnosed conditions or those not reported to health plans; consequently, some conditions may not be comprehensively identified [103]. That said, our study uses the same data that are available to health plans as they develop population health management initiatives, so the findings have potential for practical application.

## Conclusions

Using real-world administrative data, we found that sociodemographic and clinical characteristics associated with variations in the quality of care provided to Medicaid enrollees with SMI as gauged by two HEDIS measures often differ, as do the direction of the associations. We observed that patients with more complex behavioral health diagnostic profiles (i.e., those with bipolar or schizophrenia and concurrent substance use disorder or depression diagnoses, and those with concurrent bipolar and schizophrenia diagnoses) had an increased likelihood of both antipsychotic medication non-adherence (a marker of suboptimal care quality) and an increased likelihood of receiving recommended diabetes screening (a marker of higher quality care). This and other variations in the quality of healthcare received by persons with SMI that we identified can guide quality improvement and delivery system reform efforts in Medicaid plans. Our findings suggest that multidimensional approaches to improving the care quality of persons with SMI are warranted, as different mechanisms may be at play to different degrees depending on how quality is measured.

## Supplementary Information

**Additional file 1.**

## Data Availability

The data that support the findings of this study are available from Magellan Health, Inc., on behalf of the State of Florida, Agency for Health Care Administration, but restrictions apply to the availability of these data. The data were collected and analyzed during the administration and delivery of Medicaid health benefits and thus contain personal healthcare information about Florida Medicaid enrollees. By statute, such data are not publicly available. Deidentified, HIPAA-compliant data could be generated by the Magellan authors and provided to qualified researchers upon reasonable request and with permission of the State of Florida, Agency for Health Care Administration.

## Abbreviations

CPT: Current Procedural Terminology
HCPCS: Healthcare Common Procedure Coding System
HEDIS: Healthcare Effectiveness Data and Information Set
HS: High school
MCC: Magellan Complete Care
MCO: Managed care organization
MH: Mental health
MH HPSA: Designated geographic mental health professional shortage area
NCHS: National Center for Health Statistics
NCQA: National Committee for Quality Assurance
NDC: National Drug Code
PCP: Primary care physician
SAA: Adherence to Antipsychotic Medications for Individuals with Schizophrenia (HEDIS measure)
SMI: Serious mental illness
SSD: Diabetes Screening for People with Schizophrenia or Bipolar Disorder Who Are Using Antipsychotic Medications (HEDIS measure)
US: United States of America
