# Supplementary material for: Antipsychotic medication adherence and preventive diabetes screening in Medicaid enrollees with serious mental illness: an analysis of real-world administrative data
Source: BMC Health Serv Res. 2021 Jan 18;21:69. doi: 10.1186/s12913-020-06045-0 (PMC7812734; doi:10.1186/s12913-020-06045-0)
Supplement: Supplementary file 1 — Additional file 1. [file 12913_2020_6045_MOESM1_ESM.pdf]

Antipsychotic medication adherence and preventive diabetes screening in Medicaid enrollees with serious mental illness: an analysis of real-world administrative data

Additional File 1: Antipsychotic Medication List

Medication	Category
Chlorpromazine	Phenothiazine antipsychotics
Fluphenazine	
Perphenazine	
Perphenazine-amitriptyline	
Prochlorperazine	
Thioridazine	
Trifluoperazine	
Fluoxetine-olanzapine	Psychotherapeutic combinations
Thiothixene	Thioxanthenes
Aripiprazole	Long-acting injections
Fluphenazine decanoate	
Haloperidol decanoate	
Olanzapine	
Paliperidone palmitate	
Risperidone	
Aripiprazole	Other antipsychotic agents
Asenapine	
Clozapine	
Haloperidol	
Iloperidone	
Loxapine	
Lurisadone	
Molindone	
Olanzapine	
Paliperidone	
Pimozide	
Quetiapine	
Quetiapine fumarate	
Risperidone	
Ziprasidone	
